# The evolution of optimal resource allocation and mating systems in hermaphroditic perennial plants

**DOI:** 10.1038/srep33976

**Published:** 2016-09-29

**Authors:** Ya-Qiang Wang, Yao-Tang Li, Rui-Wu Wang

**Affiliations:** 1 Institute of Mathematics and Information Science, Baoji University of Arts and Sciences, Baoji, Shaanxi, China; 2 School of Mathematics and Statistics, Yunnan University, Kunming, Yunnan, China; 3 Center for Ecological and Environmental Sciences, Key Laboratory for Space Bioscience & Biotechnology, Northwestern Polytechnical University, Xi’an, 710072, China

## Abstract

By incorporating the effects of inbreeding depression (ID) on both juveniles and adults survivorship, we developed a new theoretical model for hermaphroditic perennial plants. Our model showed that the effect of the selfing rate on the evolutionarily stable strategy (ESS) reproductive allocation depends on three parameters: (1) the self-fertilized juvenile relative survivorship (SFJRS), (2) the self-fertilized adult relative survivorship (SFARS) and (3) the growth rate of self-fertilized adult, where the SFJRS is the survivorship of self-fertilized juveniles divided by the survivorship of outcrossed juveniles, and likewise for the SFARS. However, the ESS sex allocation decreases as the selfing rate increases. This relationship seems independent of the SFJRS, the SFARS, and the growth rate of self-fertilized adults. Additionally, our model showed that the complete outcrossing is an ESS when the fraction of juvenile inbreeding depression (FJID) is less than 1/2 − *τ*, where *τ* is the self-fertilized adults mortality rate caused by ID. In contrast, the complete selfing also acts as an ESS when the FJID is greater than 1/2 − *τ*. These results could explain the diversity of mating strategies and related resource allocations for plants.

In nature, around 72% species of plants possess characteristics reminiscent of both staminate (male, pollen-producing) and carpellate (female, ovule-producing) parts in the same plant. Hermaphroditic characteristics such as these allow for self-fertilization^1^,^2^,^3^. Unfortunately, self-fertilization often causes inbreeding depression (the reduced fitness in a given population as a result of breeding of related individuals). In some cases, though, self-fertilization may increase the seed set (i.e., increase female fitness gains) when pollen is limited (termed *reproductive assurance*)^4^. More importantly, self-fertilization might increase siring success (i.e., increase male fitness gains) when pollen devoted to selfing is more likely to accomplish fertilization than pollen devoted to outcrossing (termed *automatic selection advantage*)^4^,^5^. In these specific situations, self-fertilization can actually enhance the fitness of the individuals (female fitness gains plus male fitness gains) through either the sole increased female fitness gains or the male fitness gains^2^,^6^. However, the mechanism that the male or female organs should receive more allocation of resources in order to gain a fitness advantages for a given plant species is less understood.

To explore the evolution of self-fertilization in perennials, Morgan *et al*.^7^ first presented a life-history model with both overlapping generations and partial self-fertilization. Motivated by the observations that self-fertilization is comparatively more common in annual plants than among perennial plants^8^, the authors compared annual and perennial plant species and the conditions favoring self-fertilization. However, they neglected to explore how self-fertilization modifies the allocation of resources, and a similar oversight in other life-history models^1^,^6^. Harder *et al*.^9^ considered the theoretical joint effects of inbreeding depression, reproductive assurance, gamete discounting, and reproductive compensation on the evolution of hermaphroditic mating systems, specifically those of angiosperms. However, they neglected to explore how the inbreeding depression and mating systems modifies the allocation of resources^9^.

For hermaphroditic plants, self-fertilization must implement some effects on the trade-off between male and female function on resource allocation^4^,^6^,^10^. For example, if self-fertilization increases the fitness of organism through reproductive assurance, allocating more resources to female production could enhance the fitness of organisms^2^,^4^,^10^. On the contrary, if self-fertilization increases the fitness of organism through automatic selection advantage, allocating more resources to male production could enhance the fitness of organisms^2^,^4^,^10^. In either event, the fitness is increased in some way with a trade off. The increased resources in one activity must be at expense of the other. Therefore, a given organism must decide to allocate its limited resources to either the production of male or female^1^,^2^.

Zhang^6^ constructed a resource allocation model that analyzes how self-fertilization influences resource allocation for partially selfing hermaphroditic plants^6^. This model assumed that inbreeding depression only affects the survivorship of juveniles, which may only be true for annual plants. For perennial plants, instead, the inbreeding depression has been shown to potentially affect the survivorship of both juveniles and adults^2^,^6^,^11^. If inbreeding depression affects both the survivorship of juveniles and adults, the self-fertilization will influence resource allocation. This element could then be incorporated in a new and more generalized model. By incorporating these elements, we can explore several questions, under the assumption that inbreeding depression affects the survivorship of both juveniles and adults, (i) how an individual adjusts resource allocation strategy according to the level of the selfing rate, and (ii) how an individual selects might then mating strategies under different life histories.

## Methods

In our model, we only consider plants belonging to hermaphroditic perennial species, with discrete breeding seasons and overlapping generations. The individual of these species usually reach reproductive maturity after a single period (such as, 1 year) and do not alter their life-history parameters, such as survivorship and fertility^6^,^12^. We further assume that the density-dependent effect has no impact on offspring production and survival of the adult individuals. As it occurs naturally, we also assume that the resources available to the individual are limited, and that the resources can be spent only once.

**Figure 1:**
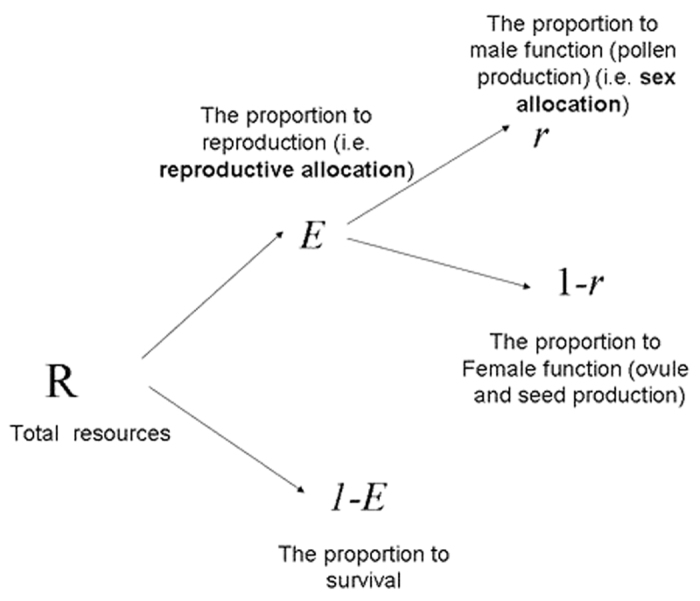
Schematic of resource allocation. *E*(= *M* + *F*) is the proportion of total resources allocated to reproduction (i.e. reproductive allocation); *r*(= *M*/*E*) is the proportion of reproductive resources allocated to male production (pollen production).

Subsequently, each individual in a monomorphic population has a total of *R* limiting resources to allocate to the three competing functions of male production, female production, and survival. Let each individual allocate a proportion *M* to the male function (pollen production), a proportion *F* to the female function (ovule and seed production) and the remaining proportion to its survival 1 − (*M* + *F*). Thus, the total reproductive allocation (the proportion of total resources allocated to reproduction) can be denoted by *E* = *M* + *F* and the remaining proportion 1 − *E* to survival. Sex allocation (the proportion of reproductive resources allocated to male production) can be denoted by *r* = *M*/*E* and the remaining proportion of reproductive resources to female production (see Fig. 1).

Since in these species inbreeding depression occurs repeatedly during several stages of their life history^6^,^12^,^13^, we assume that it affects differently juvenile and adult survivorship. Therefore, let a fraction *s* of the juveniles be selfed, and a selfed juvenile have viability *P*_*j*_*w*_*j*_ relative to a viability of *P*_*j*_ for an outcrossed juvenile, where *w*_*j*_ = 1 − *δ*_*j*_ is the survivorship of self-fertilized juveniles relative to outcrossed juveniles (termed *self-fertilized juvenile relative survivorship*) and *δ*_*j*_ is the fraction of selfed juveniles inbreeding depression^14^. Let *P*_*a*_ be an outcrossed adult survivorship and *P*_*a*_*w*_*a*_ be a self-fertilized adult survivorship, where *w*_*a*_ = 1 − *δ*_*a*_ is the survivorship of self-fertilized adults relative to outcrossed adults (termed *self-fertilized adult relative survivorship*) and *δ*_*a*_ is the fraction of self-fertilized adults inbreeding depression. Let *S* be the fraction of self-fertilized adults. Since the self-rate *s* can affect the fraction (*S*), we assume that *S* to be the function of *s*, that is *S* = *S*(*s*). Let *P*_*a*_ be adult survivorship, *f* be the number of seeds produced and *m* be the number of pollen produced be functions of their respective resource investment, that is, *f* = *f*(*F*), *m* = *m*(*M*) and *P*_*a*_ = *P*_*a*_(*E*), and *P*_*j*_ is a constant. The parameters of our model are summarized in Table 1.

We consider the fate of a rare mutant that allocates a proportion *M*′ of *R* to pollen reproduction and *F*′ to ovule and seed production. We do this following the ESS theory^15^ which determines whether the allocation pattern (*M*, *F*) is evolutionarily stable. We only consider a hermaphroditic perennial plant and the mutant with a total fitness given by the sum of the female fitness of the mutant and male fitness of the mutant. The female fitness of the mutant is the sum of the number of adult as a seed parent surviving in the next generation and the number of successful gametes as a seed parent:


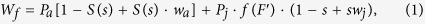


The first term of the right-hand side of Equation (1) is denoted by 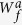. The second term is denoted by 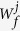. Thus, the female fitness of the mutant can be written as 
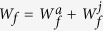
.

Similarly, the male fitness of the mutant is the sum of the number of adults as a pollen parent surviving in the next generation and the number of successful gametes as a pollen parent:


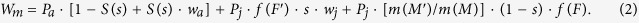


For simplicity, the first term of the right-hand side of Equation (2) is denoted by 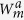. The sum of the second term and the third term is denoted by 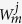. Thus, the male fitness of the mutant can be written as 
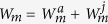
.

Notice that it is possible to have a different formulation with Equations (1) and (2), for example, Charlesworth and Charlesworth^16^. Assume that *W*_*o*_ is the total fitness gains through allocation to ovules and *W*_*p*_ is the fitness gains through allocation to pollen^16^. This usage is biologically appropriate for outcrossing species (*s* = 0), in which 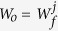 and 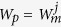. However, in most cases, they may not equal. For the sake of consistency, it is reasonable to reserve female fitness and male fitness for Equations (1) and (2), respectively, for the purpose of consistency.

From the above analysis, the total fitness *W* of the mutant is


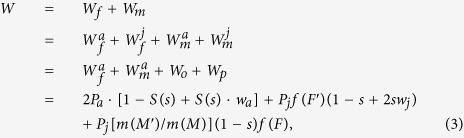


Let the common resource allocation (*M*, *F*) be evolutionarily stable, therefore, *W* is a function of with respective to *M*′ and *F*′. The total fitness *W* must attain its maximum at (*M*′, *F*′) = (*M*, *F*), that is


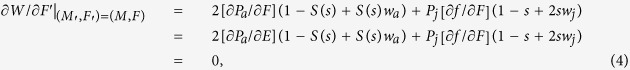



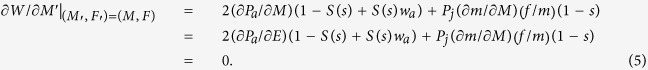


From Equations (4) and (5), we can see that these necessary conditions for an interior ESS are equal to


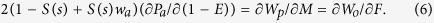


Equation (6) provides more generalized description for naturally occurring hermaphroditic perennial species than the previous results^6^,^17^. Noted *w*_*a*_ = 1, namely *δ*_*a*_ = 0, which indicates no inbreeding depression in adult, which is the similar to the Zhang^6^.

For our purposes, we use the notation of reproductive allocation (*E*) and sex allocation (*r*). Due to the definitions of reproductive allocation and sex allocation, we obtain *M*′ = *E*′*r*′, *M* = *Er*, *F*′ = *E*′(1 − *r*′) and *F *= *E*(1 − *r*). By substituting them into Equation (3) and differentiating *W* with respective to *E*′ and *r*′, we have


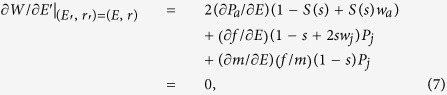



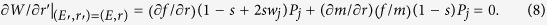


Note that


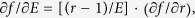


and


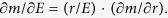


Thus, Equations (7) and (8) can be rearranged as


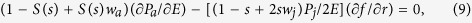



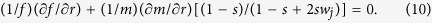


To ensure *W* attains a maximum rather than a minimum at *M* and *F* (or *E* and *r*). we also need to calculate its second derivative conditions^18^.


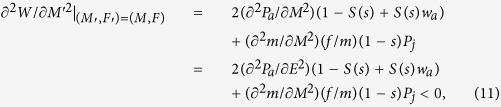



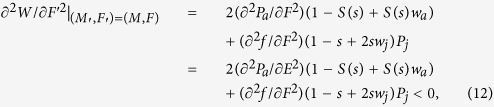



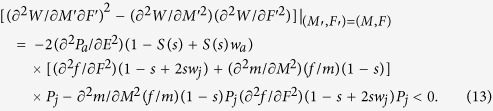


The above Equations ((4)–(5, 13) or (9)–(12)) are consistent with the general conditions for an ESS. Furthermore, from Equations (4) or (9) we obtain that the ESS reproductive allocation (*E*) will be independent of sex allocation (*r*), if and only if, female fitness gain is a linear function of resource investment (see Appendix S1). Let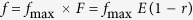, where *f*_max_ represents the maximum number of seeds produced when all available resources is spent on seed production, then Equation (9) reduces to


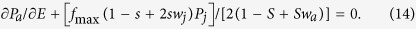


This suggests that the optimal reproductive allocation (*E*) does not depend on the sex allocation (*r*) echoing a similar result for outcrossing hermaphrodites^18^.

## Results

### ESS reproductive allocation

Given a linear function *f* (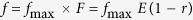) and a linear function *S* (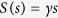, where *γ* is the growth rate of self-fertilized adult, and 0 < *γ* < 1), from Equation (11), the ESS requires that


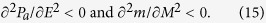


**Figure 2:**
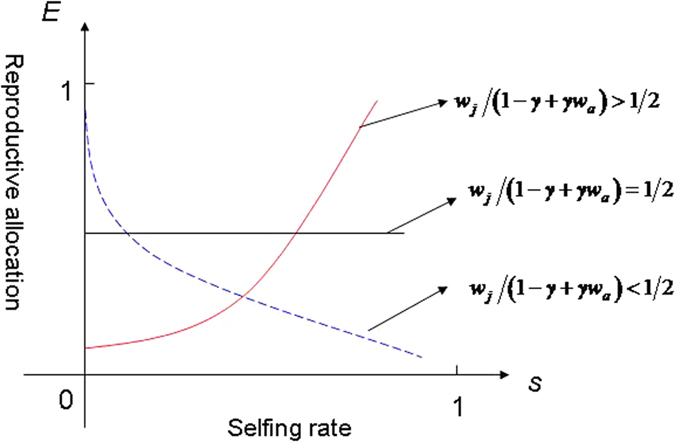
The ESS reproductive allocation (*E*) and the selfing rate (*s*). When when the ratio of *w*_*j*_ to 1 − *γ* + *γw*_*a*_ is greater than 1/2, the ESS reproductive allocation increases with increased the selfing rate (red line); when the ratio of *w*_*j*_ to 1 − *γ* + *γw*_*a*_ is less than 1/2, the ESS reproductive allocation decreases with increased the selfing rate (blue line); when the ratio of *w*_*j*_ to 1 − *γ* + *γw*_*a*_ equals to 1/2, the ESS reproductive allocation does not depend on the selfing rate (black line).

It is worth noting that the effect of the selfing rate on the ESS reproductive allocation depends on the ratio of *w*_*j*_ to 1 − *γ* + *γw*_*a*_, where *w*_*j*_ and *w*_*a*_ is the self-fertilized juvenile relative survivorship and the self-fertilized adult relative survivorship respectively, and *γ* is the growth rate of self-fertilized adult. From Equation (14), our model shows that the ESS reproductive allocation increases as the selfing rate increases when the ratio of *w*_*j*_ to 1 − *γ* + *γ w*_*a*_ is greater than 1/2, whereas the reverse is true when the ratio of *w*_*j*_ to 1 − *γ* + *γw*_*a*_ is less than 1/2. Particularly, the ESS reproductive allocation will be independent on the selfing rate when the ratio of *w*_*j*_ to 1 − *γ* + *γw*_*a*_ equals to 1/2 (see Appendix S2 and Fig. 2). The results we give here are more generalized than the result of Zhang’s^6^. In particular, the ESS reproductive allocation (*E*) increases as *s* increases if *δ*_*j*_ < 1/2, which is the special case of the ratio of *w*_*j*_ to 1 − *γ* + *γw*_*a*_ being greater than 1/2 (i.e. *w*_*a*_ = 1). Most of population genetic models in fact suggest that selfing can evolve if *δ*_*j*_ < 1/2^19^.

### ESS sex allocation

In the following context, let *f* be a linear function (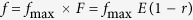) and *m* be a power function of the resource allocation investment. That is, 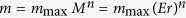, where *m*_max_ represents the maximum number of pollens produced when all available resources are spent on pollen production. Thus, the ESS sex allocation (*r*) can be solved as


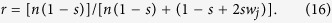


**Figure 3:**
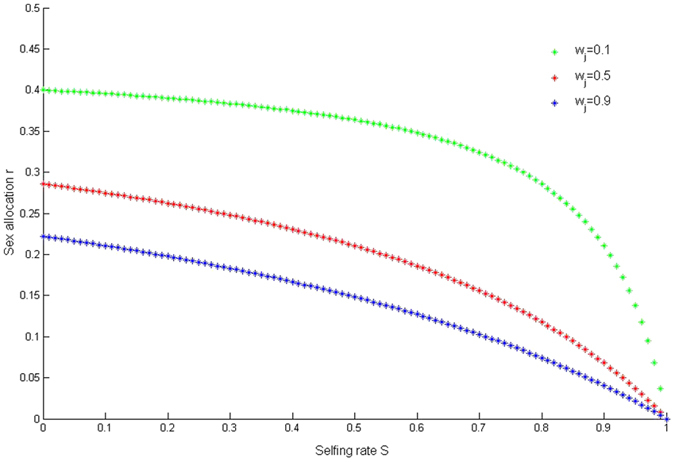
The ESS sex allocation (*r*) and the selfing rate (*s*). The Fig. 3 shows that the ESS sex allocation decreases as the selfing rate increases for any value of the survivorship of juvenile inbreeding depression (*w*_*j*_).

Equation (16) implies that sex allocation does not depend on the total reproductive allocation and does not depend on the self-fertilized adult relative survivorship. Furthermore, Equation (16) shows that the sex allocation decreases as the selfing rate increases. This relationship dose not depend on the self-fertilized juvenile relative survivorship and the self-fertilized adult relative survivorship, since 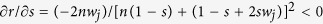. In addition, we also show that the relationship does not depend on the specific assumption of *m* being a power function of resource investment (see Appendix S3 and Fig. 3). Thus, sex allocation should generally decrease with increased selfing rate, regardless of the exact forms of the male fitness function (*m*).

### The selection of the ESS mating strategies

In the preceding analysis, we took the selfing rate as a constant, and from there we worked out the ESS reproductive allocation (*E*) and sex allocation (*r*). In what follows, we assume that the selfing rate is a variable, and considered the evolution of selfing rate. Changes in a species’ mating strategy (changes in the selfing rate, especially) may lead to the changes in resource allocation^20^. Then, the corresponding fitness for a mutant individual is


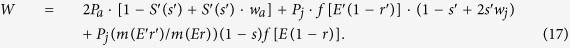


Clearly,


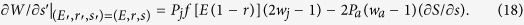


**Figure 4:**
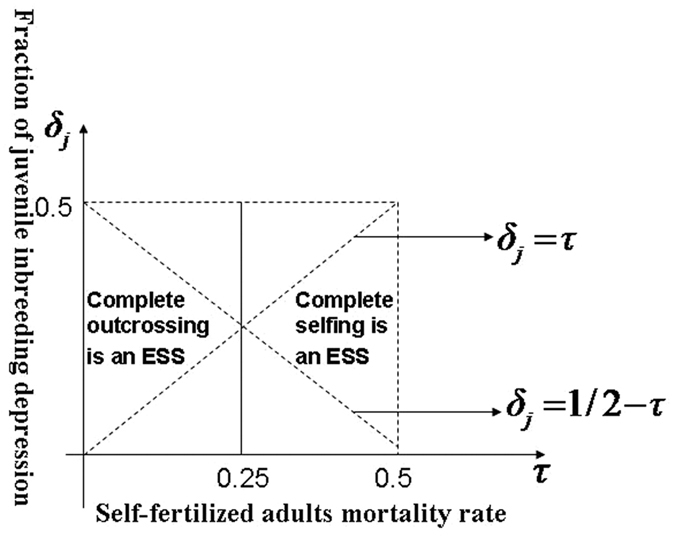
The ESS mating strategy. When the fraction of juvenile inbreeding depression (*δ*_*j*_) is less than 1/2 − *τ*, where *τ* = (*δ*_*a*_*P*_*a*_*γ*)/(*P*_*j*_*f*), the complete outcrossing is an ESS; When the fraction of juvenile inbreeding depression (*δ*_*j*_) is greater than 1/2 − *τ*, where *τ* = (*δ*_*a*_*P*_*a*_*γ*)/(*P*_*j*_*f*), the complete selfing is an ESS.

This means that a complete selfing or complete outcrossing can be evolutionarily stable, depending on the sign of 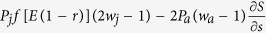. From Equation (18), when the fraction of juvenile inbreeding depression (*δ*_*j*_) is less than 1/2 − *τ*, we have 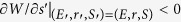. In other words, under this condition, the species chooses the complete outcrossing as the ESS (see Fig. 4). When the fraction of juvenile inbreeding depression (*δ*_*j*_) is greater than 1/2 − *τ*, we have 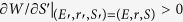, which means the complete selfing is an ESS (see Fig. 4). The parameter 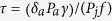 is defined here as the self-fertilized adult mortality rate caused by inbreeding depression, which is similar to the concept of *infant mortality rate* in demography^21^.

## Discussion

Existed models assumed that inbreeding depression only affects the survivorship of juveniles, and they dealt with how self-fertilization influences resource allocation^2^,^6^,^7^. This assumption is true for annual plants, but may not be ture for perennial plants, because inbreeding depression may affect the survivorship of both juveniles and adults. This may lead to a allocation for optimal resource different than custom. Simultaneously, the selection of the mating strategies may also be chosen differently. This leads to the distinct mating system found in perennial plant^6^,^7^,^11^. Accordingly, the model we describe here shows that when assuming inbreeding depression has an effect on the survivorship of both juveniles and adults, the effects of the selfing rate on reproductive allocation and the selection of mating strategy depend strongly on three parameters: (1) the self-fertilized juvenile relative survivorship, (2) the self-fertilized adult relative survivorship and (3) the growth rate of self-fertilized adult (where the self-fertilized juvenile relative survivorship is the survivorship of self-fertilized juvenile divided by the survivorship of outcrossed juvenile, and likewise for self-fertilized adult relative survivorship).

Our model shows that fluctuations in the selfing rate that leads to the variation of the ESS reproductive allocation is greatly affected by (1) the self-fertilized juvenile relative survivorship (its magnitude given by *w*_*j*_ = 1 − *δ*_*j*_), (2) the self-fertilized adult relative survivorship (*w*_*a*_ = 1 − *δ*_*a*_) and (3) the growth rate of self-fertilized adults (*γ*). On the other hand, inbreeding depression on both self-fertilized juveniles (*δ*_*j*_) and adults (*δ*_*a*_) is strongly affected by environmental conditions^22^,^23^. The impact of environmental conditions on juvenile inbreeding depression (*δ*_*j*_) and adult inbreeding depression (*δ*_*a*_), however, may differ under different circumstances^22^. If the effect of the environmental condition on adult inbreeding depression is comparatively much more severe than it is on juvenile inbreeding depression, the self-fertilized juvenile relative survivorship should accordingly be much greater than the self-fertilized adult relative survivorship (i.e. the ratio of *w*_*j*_ to 1 − *γ* + *γw*_*a*_ possibly is greater than 1/2, Fig. 2, red line). In such situation, the increase in selfing rate may raise the proportion of overall reproduction (i.e. reproductive allocation). Conversely, if the effect of a given environmental condition on juvenile inbreeding depression is much more severe than on adult inbreeding depression, the self-fertilized juvenile relative survivorship should be much less than the self-fertilized adult relative survivorship (i.e. the ratio of *w*_*j*_ to 1 − *γ* + *γw*_*a*_ possibly is less than 1/2, Fig. 2, blue line). In this situation, the increases in selfing rate may reduce the proportion of reproductive allocation. In particular, if the self-fertilized juvenile relative survivorship is equal to 1/2 times 1 − *γ* + *γw*_*a*_ (i.e. the ratio of *w*_*j*_ to 1 − *γ* + *γw*_*a*_ possibly equals 1/2, Fig. 2, black line), the ESS reproductive allocation is independent on the selfing rate.

Our model also shows that the ESS sex allocation decreases as the selfing rate increases independent from the self-fertilized juvenile relative survivorship (Fig. 3). This prediction agrees with many empirical observations made on perennial plants^2^,^11^,^24^,^25^,^26^. For instance, *Ranunculaceae* plants have reduced the allocation to male function and attractive structures such as petals that could increase the selfing rate^26^. Moreover, the ESS sex allocation decreases as the selfing rate increases, which does not depend on the assumption of *m* being a power function of resource investment (see Appendix S3). This is also in agreement with the prediction of Zhang’s^6^, though his model only considered the effect inbreeding depression on self-fertilized juveniles.

Inbreeding depression on both self-fertilized juveniles and self-fertilized adults is strongly affected by environmental conditions^22^,^23^. The fraction of juvenile inbreeding depression and adults inbreeding depression may therefore be different among species or within species under different environmental conditions. In our model, if the fraction of juvenile inbreeding depression (*δ*_*j*_) is less than 1/2 − *τ*, (where *τ* is the self-fertilized adult mortality rate caused by inbreeding depression), our model shows that the complete outcrossing is an ESS mating strategy. Conversely, the complete selfing is also an ESS mating strategy. Although no studies have shown an empirical correlation between mating strategy and the fraction of juvenile and adult inbreeding depressions, some empirical observation or experiments have implied that these inbreeding depressions can potentially affect the resulting of mating strategy adopted by these plants^6^,^7^,^25^,^27^,^28^. We recall several empirical studies showing that the mating strategy adopted by annual plants is the one of complete selfing, in the case of the juvenile inbreeding depression being less than 1/2. In our model, if the adult inbreeding depression equals to zero and the fraction of juvenile inbreeding depression (*δ*_*j*_) is less than 1/2 − *τ*, the juvenile inbreeding depression becomes less than 1/2.

While our model derived from several basic assumptions that are comparatively simpler than we would actually observe in nature, but it offers some interesting possibilities in both predicting and arriving at a greater understanding how the self-fertilized juvenile relative survivorship, the self-fertilized adult relative survivorship and the growth rate of self-fertilized adult affect ESS mating strategies and resource allocation. The theoretical nature of this model necessitates future, direct empirical demonstration of the expected correlation between mating strategy and these parameters for perennial plants. This can be facilitated by direct molecular estimates or micro-satellites^2^. Following such observations, we can construct a more refined model, mirroring more closely the strategies adopted by perennial plants. This model would consider more realistically e.g. differences in ages and life-stages, the effect of cooperation of population on resource allocation^29^,^30^,^31^. This further step would improve our understanding of how perennials allocate resources depending on the countless environmental conditions they are faced with.

## Additional Information

**How to cite this article**: Wang, Y.-Q. *et al*. The evolution of optimal resource allocation and mating systems in hermaphroditic perennial plants. *Sci. Rep.* **6**, 33976; doi: 10.1038/srep33976 (2016).

## Supplementary Material

Supplementary Information

## Figures and Tables

**Table 1:** Parameters of a perennial life history with partial self-fertilization.

Symbol	Definition
*M*	Proportion of total resource allocated to the male function (pollen production)
*F*	Proportion of total resource allocated to the female function (ovule and seed production)
*E*	Proportion of total resources allocated to reproduction
*P*_*a*_	Adult survivorship independent of mating system
*P*_*j*_	Juvenile survivorship independent of mating system
*S*	Fraction of selfed adults
*δ*_*a*_	Fraction of adult inbreeding depression
*δ*_*j*_	Fraction of juvenile inbreeding depression
*w*_*a*_	Survivorship of self-fertilized, relative to outcrossed, adults during a single period, *w*_*a *_= 1 − *δ*_*a*_
*w*_*j*_	Survivorship of self-fertilized, relative to outcrossed, juvenile during recruitment,*w*_*j *_= 1 − *δ*_*j*_
*f*	Number of seeds produced per individual
*m*	Number of pollen produced per individual
*W*	Total fitness of an individual
*W*_*f*_	The female fitness of the mutant
*W*_*m*_	The male fitness of the mutant
