# Supplementary material for: The evolution of optimal resource allocation and mating systems in hermaphroditic perennial plants
Source: Sci Rep. 2016 Sep 29;6:33976. doi: 10.1038/srep33976 (PMC5041116; doi:10.1038/srep33976)
Supplement: Supplementary Information [file srep33976-s1.pdf]

The evolution of optimal resource allocation and mating systems in hermaphroditic perennial plants

Ya-Qiang Wang[1], Yao-Tang Li[2], Rui-Wu Wang[3]*

Email:Ya-QiangWang-wangyaqiang@bjwlxy.edu.cn;Yao-TangLi-liyaotang@ynu.edu.cn; Rui-Wu Wang*-wangrw@nwpu.edu.cn

.

1.Institute of Mathematics and Information Science, Baoji University of Arts and Sciences, Baoji, Shanxi, China

2. School of Mathematics and Statistics, Yunnan University, Kunming, Yunnan, China.

3.Center for Ecological and Environmental Sciences, Key Laboratory for Space Bioscience & Biotechnology, Northwestern Polytechnical University, Xi'an, 710072, China

.

*Corresponding author: Rui-Wu Wang

E-mail: wangrw@nwpu.edu.cn

Tel: +86-029_88460397

Fax: +86-029_88460397

Supporting Information

Appendix S1 Proof that the ESS reproductive allocation (E) will be independent of sex allocation (r), if and only if, female fitness gain is a linear function of resource investment.

First, from Equations (9)

$$(1-S(s)+S(s)w_a)\left(\partial P_a/\partial E\right)-\left[(1-s+2sw_j)P_j/2E\right]\left(\partial f/\partial r\right)=0,$$

We have

$$\partial f/\partial r=\left[2E(1-S(s)+S(s)w_a)/(1-s+2sw_j)P_j\right]\left(\partial P_a/\partial E\right),$$

The right of the above equation is function of E and independent of r, therefore, it is shows that f is a linear function of r and E, that is female fitness gain is a linear function of resource investment.

Additionally, if female fitness gains is a linear function of resource investment, without loss of generality, let $f=f_{\max}\times F=f_{\max}E(1-r)$, where $f_{\max}$ is a constant.

Substituted $f=f_{\max}\times F=f_{\max}E(1-r)$ into Equations (9), we have

$$(1-S(s)+S(s)w_a)\left(\partial P_a/\partial E\right)-\left[(1-s+2sw_j)P_j/2E\right]\left(-f_{\max}E\right)=0$$

that is

$$(1-S(s)+S(s)w_a)\left(\partial P_a/\partial E\right)+(1-s+2sw_j)P_j/2f_{\max}=0.$$

The above equation only depends on E, therefore, it is shows that the ESS reproductive allocation (E) will be independent of sex allocation (r).

Appendix S2 Proof that the ESS reproductive allocation increases with increasing selfing rate if $\delta_j>\left(1+\gamma\delta_a\right)/2$.

Differentiating both sides of Equation (14) in the text with respect to s, we obtain

$$\partial\left\{\left[\partial P_a/\partial E+f_{\max}(1-s+2sw_j)P_j\right]\Big/\left[2(1-\gamma s+\gamma sw_a)\right]\right\}\Big/\partial s=$$

$$\left(\partial^2 P_a/\partial E^2\right)\left(\partial E/\partial s\right)+\left\{f_{\max}P_j\left[(1-\gamma)+\gamma w_a-2w_j\right]\right\}\Big/\left\{2\left[1-(1-w_a)\gamma s\right]^2\right\}=0$$

.

Thus

$$\partial E/\partial s = \left\{ \left[f_{\max} P_j (1-\gamma+\gamma w_a - 2w_j) \right] \Big/ \left[2\left[1-(1-w_a)\gamma s\right]^2 \right] \right\} \Big/ \left(\partial^2 P_a / \partial E^2 \right).$$

Since $\partial^2 P_a / \partial E^2 < 0$ (Equation 15), therefore, the sign of $\partial E/\partial S$ depends on the $1-\gamma+\gamma w_a - 2w_j$. It is obvious that $\partial E/\partial s < 0$ if $w_j / (1-\gamma+\gamma w_a) < 1/2$, and $\partial E/\partial S > 0$ if $w_j / (1-\gamma+\gamma w_a) > 1/2$, and $\partial E/\partial S = 0$ if $w_j / (1-\gamma+\gamma w_a) = 1/2$.

Appendix S3 Proof that the ESS sex allocation decreases with the selfing rate for any δ_j.

Differentiating Equation (10) with respect to s, we obtain

$$\partial \left\{ \partial \ln f / \partial r + (\partial \ln m / \partial r) \left[(1-s)/(1-s+2sw_j) \right] \right\} \Big/ \partial s = 0,$$

or

$$\left(\partial^2 \ln f / \partial r^2\right)\left(\partial r / \partial s\right) + \left(\partial^2 \ln m / \partial r^2\right)\left(\partial r / \partial s\right)\left[(1-s)/(1-s+2sw_j)\right]$$

$$+ \left(\partial \ln m / \partial r\right)\left[(-2w_j)/(1-s+2sw_j)\right] = 0.$$

Thus

$$\partial r/\partial s = \left\{ \frac{\left(\partial \ln m / \partial r\right)\left[2w_j / (1-s+2sw_j)^2\right]}{\partial^2 \ln f / \partial r^2 + \left(\partial^2 \ln m / \partial r^2\right)\left[(1-s)/(1-s+2sw_j)\right]} \right\}.$$

Since $\partial^2 \ln f / \partial r^2 = -\left[(1/f)(\partial f / \partial r)\right]^2 < 0$, and

$$\partial^2 \ln m / \partial r^2 = \left(\partial^2 \ln m / \partial M^2\right) E^2 = \left\{ \left(\partial^2 m / \partial M^2\right)(1/m) - \left[(\partial m / \partial M)(1/m)\right]^2 \right\} E^2$$

We obtain $\partial r / \partial s < 0$ for any δ_j if $\partial^2 m / \partial M^2 < 0$ (Equation 15).
